# Nickel Nanoparticles Induced Hepatotoxicity in Mice via Lipid-Metabolism-Dysfunction-Regulated Inflammatory Injury

**DOI:** 10.3390/molecules28155757

**Published:** 2023-07-30

**Authors:** Shuang Zhou, Hua Li, Hui Wang, Rui Wang, Wei Song, Da Li, Changlei Wei, Yu Guo, Xueying He, Yulin Deng

**Affiliations:** 1 Beijing Institute of Medical Device Testing, Beijing Center for Testing and Research of Medical Biological Protective Equipment, Beijing 101111, China; zhoushuang@bimt.org.cn (S.Z.);; 2 Beijing Institute of Technology, School of Life Science, Beijing 100081, China

**Keywords:** nickel nanoparticles, hepatotoxicity, steatosis, hepatic inflammation

## Abstract

Nickel nanoparticles (NiNPs) have wide applications in industry and biomedicine due to their unique characteristics. The liver is the major organ responsible for nutrient metabolism, exogenous substance detoxification and biotransformation of medicines containing nanoparticles. Hence, it is urgent to further understand the principles and potential mechanisms of hepatic effects on NiNPs administration. In this study, we explored the liver impacts in male C57/BL6 mice through intraperitoneal injection with NiNPs at doses of 10, 20 and 40 mg/kg/day for 7 and 28 days. The results showed that NiNPs treatment increased serum levels of alanine aminotransferase (ALT) and aspartate aminotransferase (AST) and induced pathological changes in liver tissues. Moreover, hepatic triglyceride (TG) content and lipid droplet deposition identified via *de novo* lipogenesis (DNL) progression were enhanced after NiNPs injection. Additionally, sustained NiNPs exposure induced a remarkable hepatic inflammatory response, significantly promoted endoplasmic reticulum stress (ER stress) sensors Ire1α, Perk and Atf6, and activated the occurrence of liver cell apoptosis. Overall, the research indicated that NiNPs exposure induced liver injury and disturbance of lipid metabolism. These findings revealed the public hazard from extreme exposure to NiNPs and provided new information on biological toxicity and biosafety evaluation.

## 1. Introduction

Over the past few decades, rapid advances in nanotechnology have led to the development of engineered nanomaterials for wide applications, including the chemical industry, materials manufacture and biomedicine [1,2,3,4]. It is reported that the global market value of nanomaterials is expected to grow at a compound annual growth rate (CAGR) of 14.8% from 2023 to 2030 [5]. Nickel is one of the most abundant heavy metal elements in the Earth’s crust [6]. Soluble and insoluble nickel compounds are common in nature [7]. Nickel nanoparticles (NiNPs) have extensive utilization in batteries, catalysts, textile production, ceramics and biosensors due to their distinctive characteristics such as high reactivity, high magnetism, low melting point and large surface area [8,9,10,11,12]. The universal presence of NiNPs increased their release into the environment, which brings hazards to public health and safety. For example, in 1994, a 38-year-old healthy non-smoking male inhaled NiNPs while spraying nickel onto bushes for turbine bearings, he died of adult respiratory distress syndrome (ARDS) 13 days after exposure [13]. In another case, a 26-year-old female chemist developed a severe nickel allergy when working with nickel powder with no protective measures [14]. Recent theoretical developments have revealed that occupational or non-occupational exposure to NiNPs can cause pulmonary injury, reproductive dysfunction, atherosclerosis, renal damage and even carcinogenic effects [7,15,16,17,18]. In 1990, The International Agency for Research on Cancer (IARC) recognized nickel compounds as Class I human carcinogens [6].

The liver is the largest internal organ, approximately 2–5% of the total body weight and responsible for about 13% of the body’s blood supply at any given time, and plays an important role in metabolism, protein synthesis, storage of micronutrients, production of bile and xenobiotic detoxification [19,20]. As demonstrated by previous studies, the liver is the primary component of the mononuclear phagocyte system (MPS); it is estimated that 30–99% of administered nanoparticles are sequestered and accumulated in the liver from the blood circulation system [21,22]. The flow rate of nanomaterials reduces 1000-fold when they interpenetrate the liver, and 7.5 times nanomaterials are accumulated through the acquisition of Kupffer cells, immune B cells and hepatic sinusoidal endothelial cells [23,24]. The excessive deposition of nanomaterials could induce adverse hepatic impacts including metabolic enzyme activity interference, oxidative stress, inflammation response, impaired lipogenesis, hepatocyte apoptosis and fibrosis [25,26]. Thus, hepatic nanotoxicology became a research area of interest in biological safety evaluation and biomedical applications.

NiNPs in the bloodstream are mainly detained by the liver [27,28,29]. Recent reports indicated that nickel and nickel-containing nanoparticles exhibited liver injury in vivo and in vitro. Ajdariand and co-authors demonstrated that intraperitoneal injection with 50 nm NiNPs (75 ppm) significantly induced liver pathological changes in mice and initiated cycle arrest with apoptosis in HepG2 [28,30,31]. Nickel oxide nanoparticle (nano NiO) regulated the activation of inflammation response to induce oxidative-stress-mediated liver toxicity in rats [32]. Nickel ferrite nanoparticles (NiFe_2_O_4_) provoked cell survival decrease in HepG2 through generating excessive ROS [33]. At present, the processes of these manifestations have not been completely elucidated, so it is a critical requirement to further explore the potential molecular mechanisms and development process of NiNPs effects on hepatotoxicity. Based on the confirmed research findings, we proposed the reasonable hypothesis that massive NiNPs exposure is able to initiate homeostatic imbalance of the energy metabolism, and persistent inflammation response induced toxicity and damage effects in the liver. In this study, we intraperitoneally injected C57/BL6 mice with 50 nm NiNPs with concentrations of 10, 20 and 40 mg/kg/day for 7 and 28 days to investigate lipid metabolism, hepatic inflammatory reaction, endoplasmic reticulum stress (ER stress) and liver cell apoptosis effects in the liver after NiNPs treatment.

## 2. Results

### 2.1. Physicochemical Characterization of NiNPs

**Figure 1:**
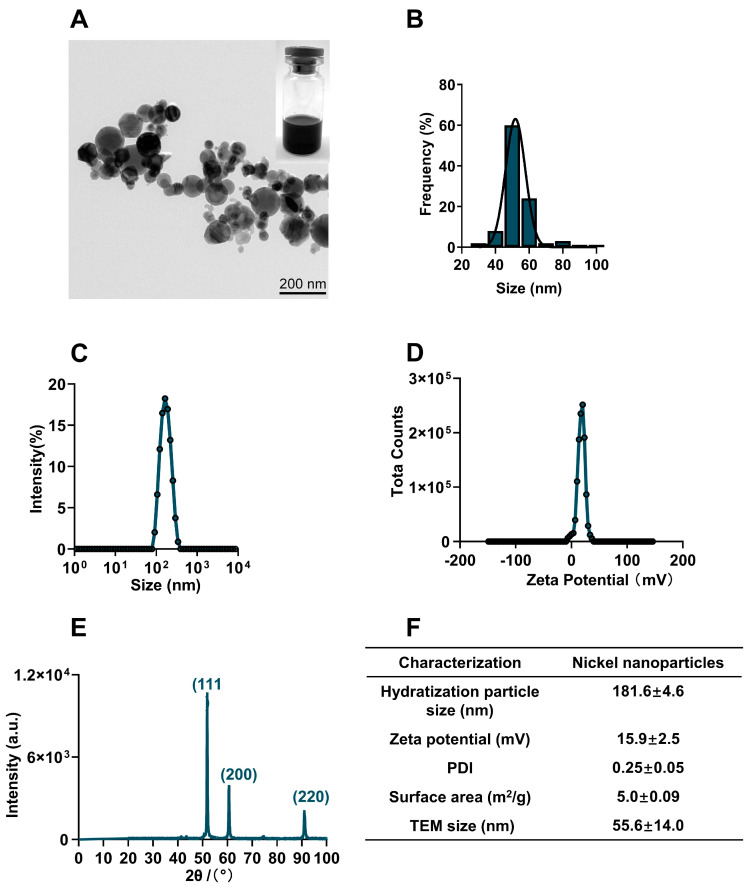
Physicochemical characterization of NiNPs. (**A**) TEM images showed the morphology of NiNPs. Scale bar, 200 nm. (**B**) Gaussian distribution of NiNPs diameter. (**C**,**D**) Hydrodynamic size distribution and surface charge of NiNPs measured by DLS. (**E**) X-ray diffraction (XRD) patterns of NiNPs. (**F**) Specific information on the NiNPs used in this research.

The commercial NiNPs powder was characterized in terms of physical properties, particle diameter, chemical composition, crystal structure, hydrodynamic size, surface charge and surface area. Transmission electron microscope (TEM) images showed NiNPs well dispersed in Milli-Q water and the statistical average diameter was 55.8 ± 14.0 nm (Figure 1A,B). The hydrodynamic size of NiNPs in Milli-Q water was observed to be 181.6 ± 4.6 nm with a narrow distribution of size (PDI: 0.25 ± 0.05); meanwhile, the zeta potential was detected as 15.9 ± 2.5 mV (Figure 1C,D). The X-ray diffraction (XRD) pattern showed typical diffraction peaks of the cubic phase. The strong and narrow diffraction peaks indicated that the powder had a good crystal structure (Figure 1E). The BET analysis results showed that the specific surface area was 5.0 ± 0.09 m^2^/g (Figure 1F). In summary, NiNPs had good dispersibility and stability for administration.

### 2.2. NiNPs Treatment Induced Liver Damage and Pathological Changes in Mice

**Figure 2:**
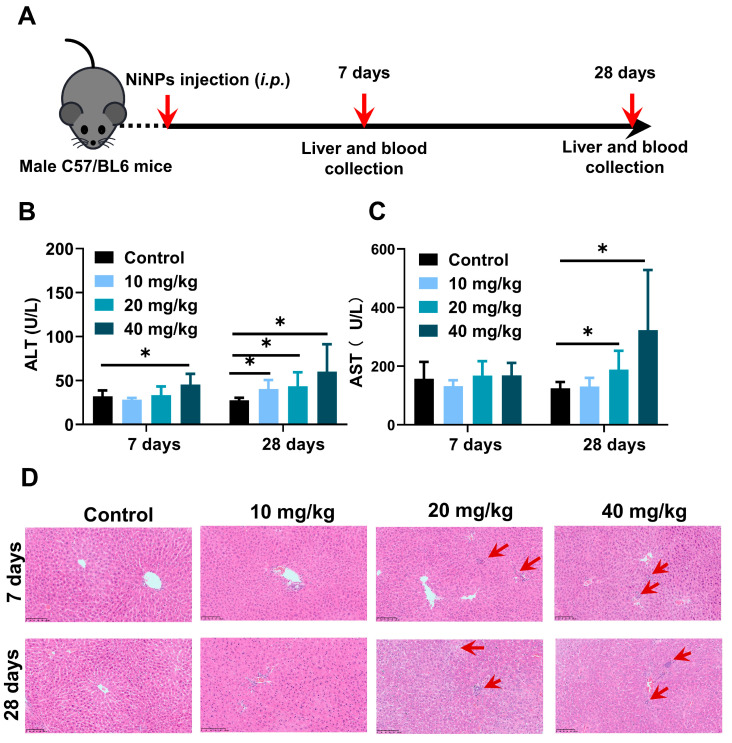
NiNPs exposure increased hepatic damage in mice. (**A**) Schematic illustration of experimental design. C57/BL6 mice were intraperitoneally injected (i.p) with NiNPs at dosages of 10, 20, 40 mg/kg/day for 7 and 28 days. (**B**,**C**) Serum ALT, AST levels of mice. (**D**) H&E results of the histological changes. Red arrow: macrophage infiltration; scar bar, 100 μm. Each bar represents mean ± SD. (*n* = 6, * *p* < 0.05 compared to control group).

For the purpose of evaluating the hepatic effects on NiNPs-treated mice, NiNPs were intraperitoneally injected into the mice at doses of 10, 20and 40 mg/kg/day (body weight) for 7 and 28 days (Figure 2A). The levels of ALT and AST exhibited significant increases in high-concentration-treated mice after continuous 28-day exposure compared with the control group (Figure 2B,C). Moreover, the results of liver hematoxylin and eosin (H&E) staining displayed obvious inflammatory cell infiltration and hepatic vacuolation, suggesting the topical inflammatory response and hepatocyte lipid deposition (Figure 2D). Together, all these data confirmed that NiNPs exposure efficiently initiated liver damage and metabolism disturbance in C57/BL6 mice, indicating the possible pathogenic mechanisms of NiNPs-caused hepatotoxicity.

### 2.3. NiNPs Treatment Induced Lipid Deposition in Mice Liver

**Figure 3:**
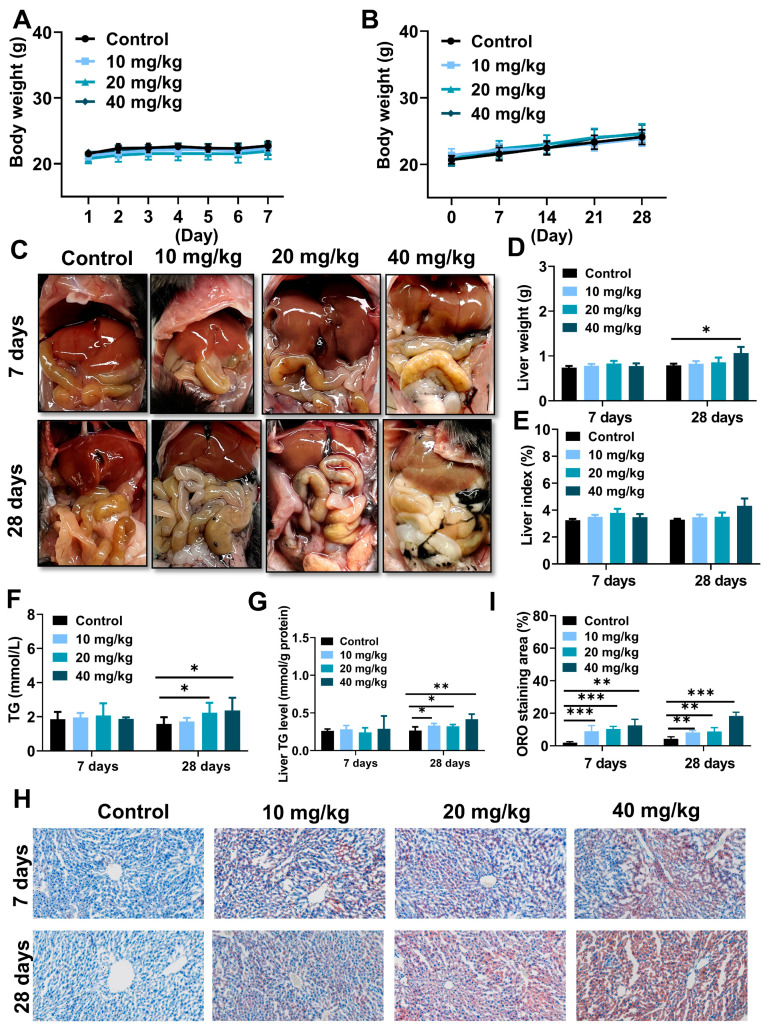
NiNPs exposure induced liver steatosis in mice. (**A**,**B**) Average body weight of control and NiNPs-treated mice. (**C**) Brightfield images for the color of NiNPs-treated and control mice liver. (**D**,**E**) Liver weight and liver index. (**F**,**G**) Serum and hepatic TG levels in mice. (**H**,**I**) Mice hepatic steatosis was detected using ORO staining and statistical analysis of lipid droplet deposition in mice. Scar bar, 50 μm. (*n* = 6, * *p* < 0.05, ** *p* < 0.01, *** *p* < 0.001 compared to control group).

As represented in Figure 3A,B, there were no remarkable differences between NiNPs-treated and control groups in terms of the body weight of the mice. Notably, NiNPs administration leads to various manifestations of lipid accumulation and even steatosis, reflected in the weakened color of the liver (Figure 3C) and increased liver weight (Figure 3D). In addition, triglyceride (TG) levels in serum and liver tissues were elevated (Figure 3F,G), and Oil Red O (ORO) images showed that lipid deposition activated hepatic steatosis (Figure 3H,I). However, the liver index of the NiNPs treatment groups exhibited no significant differences (Figure 3E) compared with the control mice. The findings revealed that NiNPs injection evoked disorders of hepatic lipid metabolism, especially in high-concentration exposure group mice. 

**Figure 4:**
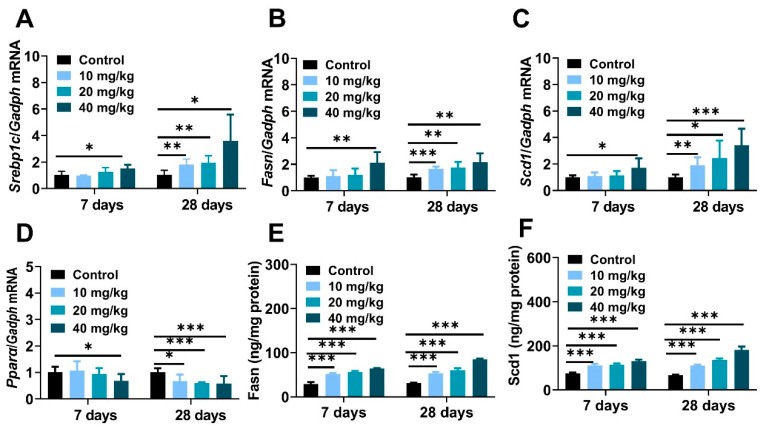
NiNPs exposure enhanced liver Srebp-1c-mediated DNL in mice. (**A**–**C**) Hepatic mRNA expression of genes related to DNL and (**D**) fatty acid oxidation. (**E**,**F**) Liver contents of *Fasn* and *Scd1* were tested by ELISA experiments. (*n* = 6, * *p* < 0.05, ** *p* < 0.01, *** *p* < 0.001 compared to control group).

To a large extent, nanoparticle-induced imbalance of hepatic lipid metabolism owing to interference of Srebp1c-initiated *de novo* lipogenesis (DNL) [34,35]. The mRNA expressions of the *Srebp1c* regulator and its downstream lipogenic genes containing fatty acid synthase (*Fasn*) and stearoyl coenzyme A desaturase 1 (*Scd1*) were obviously increased (Figure 4A–C), and the molecular abundance of hepatic *Fasn* and *Scd1* was also identically demonstrated through ELISA experiments (Figure 4E,F). In contrast, real-time PCR analysis represented the inhibitory effect on peroxisome proliferator-activated receptor α (*Pparα*) enzyme in long-term exposure mice (Figure 4D). In summary, all of these data provided proof that NiNPs treatment disturbs homeostasis of liver lipid synthesis via strengthening the progress of DNL, which suggested the potential molecular principles on NiNPs-induced liver lipotoxicity in mice.

### 2.4. NiNPs Treatment Induced Liver Inflammation in Mice 

**Figure 5:**
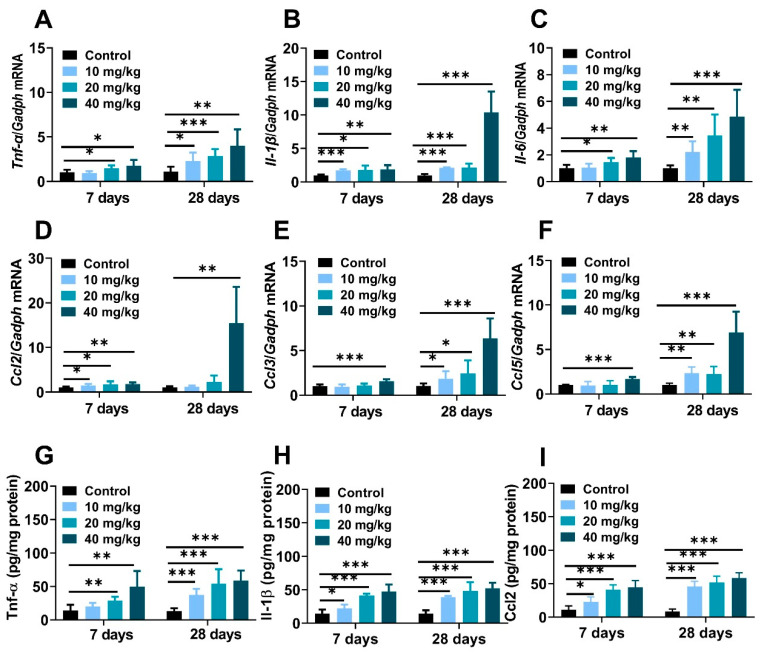
NiNPs exposure induced liver inflammation in mice. (**A**–**C**) Hepatic mRNA expressions of the pro-inflammatory cytokines and (**D**,**E**) chemokines in mice. (**G**–**I**) Hepatic levels of *Tnf-α*, *Il-1β* and *Ccl2* measured using ELISA. (*n* = 6, * *p* < 0.05, ** *p* < 0.01, *** *p* < 0.001 compared to control group).

The massive production of cytokines and chemokines were pivotal trigger factors in nanoparticle-induced inflamed liver [36]. Compared to the control mice, hepatic mRNA expression of proinflammatory cytokines, including tumor necrosis factor-alpha (*Tnf-α*), interleukin-6 (*Il-6*), interleukin-1β (*Il-1β*) and chemokine factors monocyte chemoattractant protein-1 (*Ccl2/Mcp-1*), macrophage inflammatory protein-1α (*Ccl3/Mip-1*), the regulation of activation expression and secretion by normal T Cells (*Ccl5/Rantes*) were significantly increased after NiNPs injection (Figure 5A–F). Meanwhile, the results of the ELISA tests explained that NiNPs administration increased protein levels of *Tnf-α*, *Il-1β*, and *Ccl2* in mice (Figure 5G–I). Apparently, NiNPs administration can induce a hepatic inflammatory response in mice livers.

### 2.5. NiNPs Treatment Induced Endoplasmic Reticulum Stress and Apoptosis in Mice Liver

**Figure 6:**
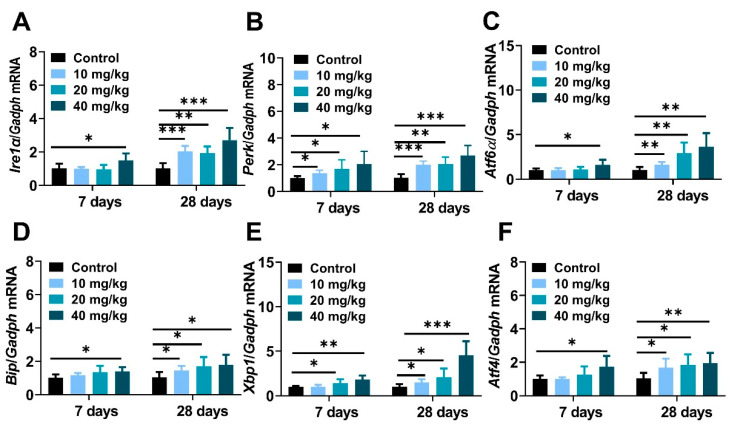
NiNPs injection activated endoplasmic reticulum stress in mice liver. (**A**–**F**) The expression of ER stress biomarkers *Bip*, *Ire1α*, *Perk*, *Atf6*, *Xbp1s* and *Atf4*. (*n* = 6, * *p* < 0.05, ** *p* < 0.01, *** *p* < 0.001 compared to control group).

It is well known that excessive nanoparticles exposure can mediate endoplasmic reticulum dysfunction and cell apoptosis in animals and cells [37,38]. This study aimed to explore the underlying mechanisms of NiNPs-induced hepatotoxicity in mice, and the gene expressions regarding endoplasmic reticulum stress (ER stress) were examined. The data showed that the mRNA levels of typical ER stress regulatory elements inositol-requiring transmembrane kinase/endonuclease-1 (*Ire1α*), PKR-like ER kinase (*Perk*), activating transcription factor 6 (*Atf6*), immunoglobulin heavy-chain-binding protein (*Bip*), X-box-binding protein-1(*Xbp1*), and activating transcription factor 4 (*Atf4*) were obviously increased in NiNPs-treated mice compared with the DPBS-injected groups (Figure 6A–C).

**Figure 7:**
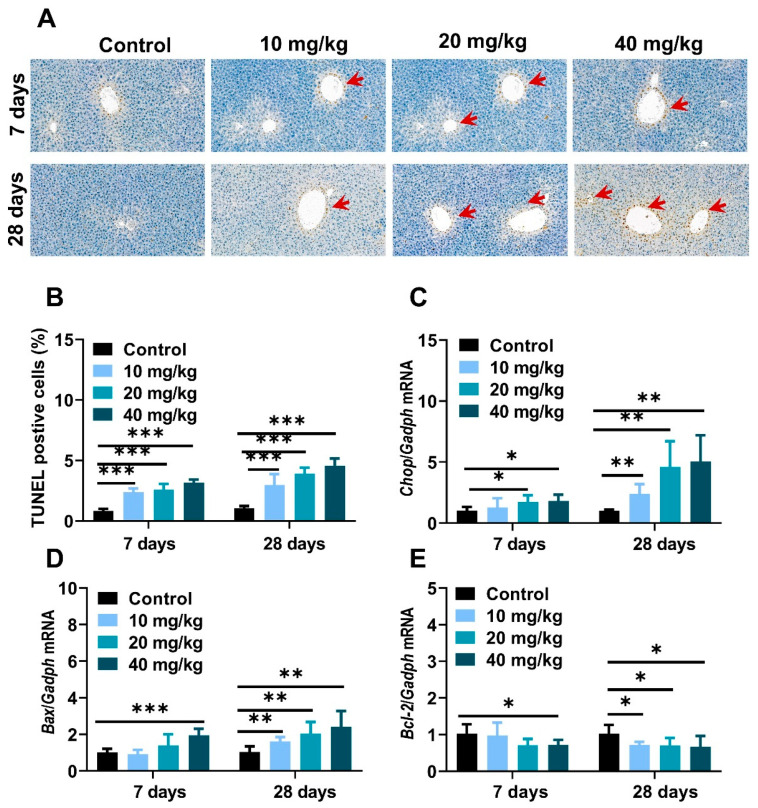
NiNPs exposure induced cell apoptosis in mice. (**A**,**B**) Representative TUNEL staining and data statistics of hepatocyte apoptosis in liver. Red arrows: apoptotic liver cells, scar bar, 50 μm. (**C**–**E**) Genes expression of *Chop*, pro-apoptosis protein *Bax* and anti-apoptosis protein *Bcl-2* in mice were examined through real-time quantitative PCR. (*n* = 6, * *p* < 0.05, ** *p* < 0.01, *** *p* < 0.001 compared to control group).

To further investigate the pathological development and mechanism in NiNPs-affected mice liver, a TUNEL assay was used to evaluate hepatocyte apoptosis. As shown in Figure 5, positive liver cells were slightly increased in low-concentration exposure groups and stronger in 20- and 40 mg/kg-treated mice compared to the control mice (Figure 7A,B). Moreover, C/Ebp-homologous protein (*Chop*), Bcl-2 associated X protein (*Bax*), and B-cell lymphoma-2 (*Bcl-2*) gene expressions were measured to demonstrate the basic pathogenic mechanisms of apoptosis (Figure 7C–E). The results exhibited the upregulated mRNA levels of ER stress sensors and apoptosis-correlated factors in NiNPs-administrated mice livers, which were similar to the TUNEL staining results. Taken together, we found that NiNPs exposure stimulated ER stress and then mediated the downstream hepatocyte apoptosis signal pathway in mice.

## 3. Discussion

Nickel is known to have a dual nature in biological systems. Micronutrient nickel plays an essential role in catalyzing several metalloenzymes in bacteria and lower eukaryotes, which was recognized as a “possibly essential element” as early as the 1970s for animals and humans [39]. On the contrary, the combustion of nickel-containing fossil fuels, nickel-manufacturing industries and the utilization of nickel products has induced the excessive release of nickel compounds and nanoparticles into the environment, leading to hazardous health effects on organisms [39,40]. Previous studies have demonstrated that nickel nanoparticles have more intense toxicity and stronger sensitization than microparticles [29,41]. The liver is the major biological filtration barrier organ responsible for the sequestration and biodegradation of exogenous substances, medicines and nanomaterials [37]. It is well known that sustained and excess metallic nanoparticles exposure induces severe liver injury [26,42]. The findings of our study showed that continuous NiNPs administration at doses of 10, 20, and 40 mg/kg/day for 7 and 28 days significantly increased hepatic steatosis, liver inflammatory responses, endoplasmic reticulum stress (ER stress) and apoptotic liver cells in male C57/BL mice, suggesting the molecular fundamentals and pathogenic progression of NiNPs-induced hepatotoxicity.

As we know, nanomaterial-mediated body weight reduction is an important biomarker of nanotoxicity in animals. Nevertheless, we found that the body weight showed no statistical differences in control and NiNPs-treated mice. In recent reports, low, medium and high concentrations of 90 nm NiNPs-induced male BALB/c mice reproductive toxicity, but no significant differences in body weight were observed compared to the control group via the intragastric approach [43]. Similarly, Kong and its colleagues found that intratracheal instillation NiNPs could provoke remarkable spermatogenic cell damage and slightly decrease mice body weight, yet the overall trend related to body weight is upward, and statistical differences were not measured during the whole experiment period [15]. Our results were consistent with these previous studies. Body weight loss is a centralized expression of metabolic system dysfunction and chronic disease. Within our exposure periods and dosages, a fraction of NiNPs may aggregate after intraperitoneal administration, which could alleviate its hepatotoxicity, so to some degree, compensatory and detoxification were still functioning in the healthy mice livers. In the following study, we will implement more explorations in response to this fact.

In our research, intraperitoneally injected (i.p.) NiNPs enhanced liver pathological characteristics, as well as serum ALT and AST levels, and elevated hepatic TG contents revealing impaired basic functions and excess lipid droplet deposition in mice livers [44]. To deeply explore the principles of NiNPs-stimulated lipotoxicity, ORO staining was detected, and we concluded that NiNPs administration induced liver steatosis in normal mice. Then, crucial genes related to the hepatic lipid metabolism process were examined through real-time fluorescence quantitative PCR. Theoretically, the liver accounts for about 26% of circulating fatty acid flux in steatohepatitis patients; SREBP1c-mediated DNL plays an important role in the biosynthesis of free fatty acids and triglycerides in the liver [44,45]. Our data showed that the NiNPs treatment upregulated the mRNA expressions of *Srebp1c*, *Fasn* and *Scd1*, which indicated abnormal hepatic lipid synthesis in mice. *Pparα* is mainly responsible for assimilating carbohydrates and lipids and activating fatty acid oxidation in lipolysis. In this work, we found the inhibition of *Pparα* gene expression, in particular in long-term exposed mice [46]. We previously proved that the administration of gold nanoparticles (GNPs) significantly upregulated pivotal gene mRNA expression of DNL in normal ICR mice [25]. In addition, polyethylene glycol-modified GNPs (PEG-GNPs) increased lipogenesis and decreased the β-oxidation of fatty acids, resulting in aggravated hepatic steatosis and more severe liver inflammation in MCD-induced nonalcoholic steatohepatitis (NASH) mice [37]. In addition, iron oxide nanoparticles (IONPs) exacerbated lipid accumulation in nonalcoholic fatty liver disease (NAFLD) mice through BMP-SMAD motivated iron overload effect [34]. Therefore, in our study, NiNPs injection evoked lipid synthesis disorder and liver fatty degeneration due to enhanced DNL progression and attenuated lipolysis in mice.

Hepatic inflammation is regarded as a key driver of the pathogenesis and development of chronic liver diseases and hepatotoxicity [47,48]. Excessive and sustained exposure to nanomaterials stimulated oxidative stress, hepatocyte injury and liver inflammatory responses [42]. Male mice administrated with NiNPs via oropharyngeal aspiration caused greater mRNA induction of *Il-6* and *Ccl2* in the liver [18]. ICR mice injected with mesoporous silica nanoparticles (MSNs) for 10 days showed significantly increased hepatic *Il-1β*, *Tnf-α*, and *Il-6* [49]. In the development process of liver damage, *Tnf-α* is hypersensitive and contributes to the activation of immune cells and hepatic stellate cells (HSC) [50]. *Il-1β* is mainly secreted from macrophages; therefore, macrophage infiltration is an important pathological marker of inflammatory reaction, and the hyperproduction of *Il-1β* accelerated spontaneous fibrosis in the liver [50]. *Il-6* can be produced through hepatic parenchymal cells in response to chronic liver diseases and hepatitis B virus (HBV) infection [51]. The primary function of chemokines (*Ccl2*, *Ccl3*, *Ccl5*) is to participate in wound-healing response and recruitment of immune and non-immune cells to inflamed areas [50]. In this study, the expressions of the involved inflammatory genes, including *Tnf-α*, *Il-6*, *Il-1β* and *Ccl2*, *Ccl3*, *Ccl5*, were increased, indicating that consecutive treatment of NiNPs is closely associated with inflammation in mice liver, which was promoted via lipid deposition-mediated liver injury.

The endoplasmic reticulum (ER) is an intracellular organelle and plays a critical role in molecule protein production, protein collapse, calcium ion homeostasis and lipid synthesis [52]. Endoplasmic reticulum (ER) stress is known as unfolded protein response (UPR), which is due to the accumulation of misfolded proteins. ER stress is characterized by the activation of three sensors, including IRE1α, PERK, and ATF6α. BIP is an ER-resident chaperone protein encoded by heat shock protein family A (Hsp70). In liver disease, misfolded proteins are detained and accumulated in the ER lumen, competitively combining with BIP, leading to its separation from the mentioned UPR sensors [53]. IRE1α is a highly conserved signal sensor, and when dissociated from BIP, IRE1α mediates the autophosphorylation of spliced Xbp1 protein (Xbp1s), and then Xbp1s regulates ER-associated degradation (ERAD) and subsequent cascade reactions. PERK is a kind of transmembrane protein, and in the ER stress state, PERK substrate eukaryotic translation initiation factor 2α (elf2α) is phosphorated, activates the translation of transcription factor 4 (ATF4), and then ATF4 is transferred to the nucleus, regulating the transcription of cell death genes. Unanchored BIP releases ATF6α, and then ATF6α is cleaved into ATF6p50 in the Golgi apparatus to regulate the effects of ERAD [54]. Various pieces of the literature indicated that, in a pathological state, chronic ER stress regulated the gene transcription of downstream spliced Xbp1s and ATF4, etc., by stimulating c-Jun N-terminal kinase (c-JNK), AMP-activated protein kinase (AMPK) and ROS, to achieve hepatic inflammatory response and hepatocyte apoptosis [55]. Chop is considered the key regulator in the ER stress-mediated apoptosis pathway. Bcl-2 is a classic antiapoptotic factor that can be downregulated by UPR-generated Chop [55]. Previous reports have demonstrated that the intratracheal instillation of 20 nm silver nanoparticles (0.1 and 0.5 μg /g BW) for 8 and 24 h increased mRNA and molecular protein levels of IRE1α, PERK, Xbp1s, and Bip in ICR mice liver. Furthermore, 20–40 nm copper oxide nanoparticles (Nano-CuO) treatment caused oxidative stress and triggered the ER stress pathway in male rat livers [38]. TUNEL staining and real-time qPCR data showed that the intraperitoneal administration of NiNPs significantly increased the amount of apoptotic-positive liver cells and enhanced mRNA expression levels of ER stress sensors *Ire1a*, *Perk*, and *Atf6a*, key regulating factors *Bip*, *Xbp1s*, and *Atf4*, apoptosis-associated genes *Chop*, *Bcl-2* and *Bax* in our works. These findings exhibited that NiNPs injection resulted in the impaired function of ER and programmed cell death through persistent inflammation and lipogenesis disturbance in mice liver. However, it is necessary to admit that more details and verifications need to be confirmed in subsequent research.

## 4. Conclusions

**Figure 8:**
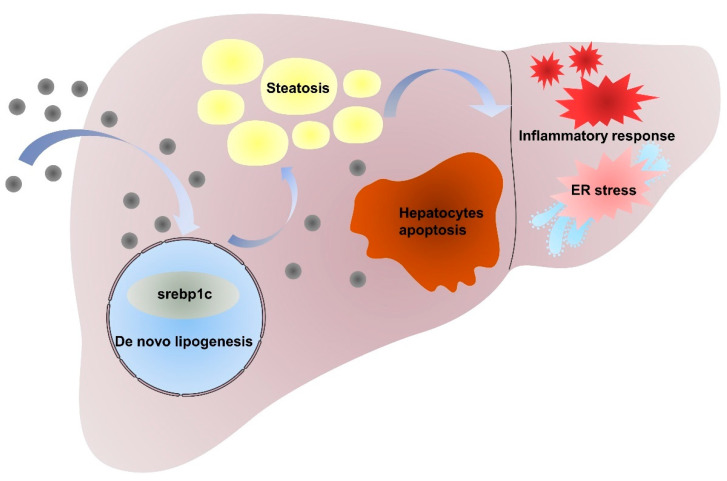
Mechanism of NiNPs treatment effects on liver injury in mice.

In conclusion, our studies demonstrated that the intraperitoneal administration of NiNPs at doses of 10, 20, and 40 mg/kg/day (body weight) for 7 and 28 days in male C57/BL6 mice has significantly promoted the progression of hepatic lipid accumulation due to *de novo* lipogenesis (DNL) dysfunction. Furthermore, NiNPs administration triggered hepatic inflammation responses in mice livers. The chronic secretion of inflammatory cytokines and chemokines induced ER stress and hepatocyte apoptosis (Figure 8). These results, to a certain extent, verified the hypothesis that NiNPs injection induced impaired lipogenesis and liver damage manifested by liver steatosis and inflammation response. The findings further our understanding of the molecular mechanisms of NiNPs-caused hepatotoxicity in mice, in addition to supplementing new thoughts for biotoxicology assessment.

## 5. Materials and Methods 

### 5.1. Physicochemical Characterization of NiNPs

NiNPs powder was purchased from Shanghai Chao Wei Nanotechnology Co., Ltd. (Shanghai, China) and characterized by the manufacturer as nanoparticles with 99.9% metal purity. The morphological characteristics and particle size of the NiNPs were measured using a transmission electron microscope (TEM, FEI Tecnai G2 F30, Hillsboro, OR, USA). The chemical composition and crystal structure were detected through X-ray diffraction (XRD, Bruker D8 Advance, Billerica, MA, USA). Dynamic light scattering (DLS, Malvern Panalytical Zetasizer Nano ZS90, Malvern, UK) was used to determine the average hydrocyanic size, zeta potential and polydispersity index (PDI). The surface area was detected using nitrogen sorption isotherms (Micromeritics, TriStar II 3020 3.02, Brentwood, TN, USA). Prior to exposure, NiNPs were suspended in phosphate-buffered saline (Solarbio Life Science, Beijing, China) with 0.1% pluronic F-68 (Sigma, Cream Ridge, NJ, USA) and ultrasonic dispersed for 30 min, and subsequently vortexed immediately before each experiment [18,56].

### 5.2. Animals and Treatments Design 

Male C57/BL6 mice (8–10-week-old, 20 ± 2 g) were obtained from Beijing Vital River Laboratory Animal Technology Co., Ltd. (Beijing, China). The mice were placed in an air-conditioned room (temperature of 20 °C ± 2, relative humidity of 60 ± 10%) under a regular 12 h light/dark cycle. The mice were allowed ad libitum access to rodent chow and water during the entire experiment period. The mice were exposed to NiNPs via intraperitoneal injection (i.p.) at concentrations of 10, 20 and 40 mg/kg/day (n = 6 per group) for 7 and 28 days. The mice injected with 200 μL phosphate-buffered saline (DPBS) containing 0.1% pluronic F-68 (Sigma) were utilized as the control group [56].

### 5.3. Serum Biochemical Indexes Analysis

After sacrificing mice, the blood was collected and centrifugated at 3000 rpm/min for 15 min at 4 °C. ALT, AST and TG levels in the mice serum were detected using a 7180 clinical analyzer (Hitachi, Tokyo, Japan).

### 5.4. Histopathological Analysis

The liver samples were fixed in 4% paraformaldehyde at room temperature and then embedded in paraffin. Tissue fragments were cut into 4 μm thick sections using a Leica RM2255 Fully Automated Rotary Microtome (Leica, Wetzlar, Germany) and stained with hematoxylin and eosin (H&E). Hepatic inflammation was evaluated by recording inflammatory cell infiltration and steatosis. 

### 5.5. TUNEL Staining Assay

Collected liver tissues were fixed in 4% paraformaldehyde and then embedded in paraffin. Next, the fragments were sectioned into 5 μm segments using Leica RM2016 Microtome (Leica, Wetzlar, Germany) and then attached to glass slides for heat treatment for 2 h at 60 °C. After dewaxing with xylene, the slices were cleaned in absolute ethanol (5 min), 90% ethanol (2 min), 70% ethanol (2 min), and deionized water (2 min). Finally, the slices were treated with a TUNEL kit (Sigma-Aldrich, Burlington, MA, USA) and DAB (Sigma-Aldrich) agent for color visualization. The negative nucleus was blue dyed with hematoxylin, and the positive apoptosis nucleus was brown under DAB.

### 5.6. Oil Red O Staining

For the measurement of lipid accumulation in mice livers, the tissues were fixed in OCT. The 7 μm frozen liver tissue sections were cut using a CRAFTEK CR-601ST Semi-motorized Rotary Microtome (CRAFTEK, Wuhan, China), stained with Oil Red O agent (Sigma-Aldrich) for 20 min and then the nucleus was counterstained with hematoxylin for 10 min at room temperature. Lipid droplets in the liver tissues were observed as being red.

### 5.7. Detection of Mice Liver TG Content

Liver tissues were homogenized, and the content of hepatic TG was tested using a commercial kit according to the manufacturer’s instructions (Nanjing Jiancheng Bioengineering Institute, Nanjing, China).

### 5.8. RNA Extraction and Quantitative RT-PCR 

Total RNA was extracted from 30 to 50 mg frozen liver tissues using TRIzol reagent (Invitrogen, Waltham, MA, USA) in accordance with the manufacturer’s instructions. RNA was quantified with NanoDrop^TM^ 2000 Spectrophotometer (ThermoFisher Waltham, MA, USA). In total, 5 μg of RNA was reverse-transcribed to cDNA following 20 μL mixture system: 5 μL total RNA, 10 μL 2 × ES Reaction Mix, 1 μL RT/RI Enzyme Mix, 1 μL Anchored Oligo (dT)_18_, 3 μL RNase-free water according to cDNA Reverse Transcription Kit (TransGen Biotech, Beijing, China). The determination system was performed on Applied Biosystems 7500 Real-Time PCR System (ThermoFisher. The reaction procedures are as follows: 94 °C for 30 s, 94 °C for 5 s with 40 cycles and 60 °C for 30 s. The PCR primers are listed in Table S1.

### 5.9. Elisa Experiment

Liver tissues (100 mg) were homogenated and then centrifuged at 5000× *g* for 10 min at 4 °C, and the levels of *Tnf-α*, *Il-1β* and *Ccl2* were measured using commercial EILSA kits (Elabscience, Wuhan, China). The content of hepatic *Fasn* and *Scd1* was detected through the use of kits (Shanghai YuBo Biological Technology, Shanghai, China).

### 5.10. Statistical Analysis

All data were represented as mean values ± standard deviation (SD) of 6 animals per groups by GraphPad Prism software (version 9.0, GraphPad Software, Inc., San Diego, CA, USA). The statistical significance was calculated using *t*-test or one-way analysis of variance (ANOVA). The *p*-value less than 0.05 was considered significant.

## Data Availability

Data will be made available upon request.

## Supplementary Materials

The following supporting information can be downloaded at: https://www.mdpi.com/article/10.3390/molecules28155757/s1, Table S1: The primer sequences used in this work.

## Sample Availability

Samples of the compounds are not available from the authors.

## References

[1] Baig N., Kammakakam I., Falath W. (2021). Nanomaterials: A review of synthesis methods, properties, recent progress, and challenges. Mater. Adv..

[2] Pelaz B., Alexiou C., Alvarez-Puebla R.A., Alves F., Andrews A.M., Ashraf S., Balogh L.P., Ballerini L., Bestetti A., Brendel C. (2017). Diverse Applications of Nanomedicine. ACS Nano.

[3] Khin M.M., Nair A.S., Babu V.J., Murugan R., Ramakrishna S. (2012). A review on nanomaterials for environmental remediation. Energy Environ. Sci..

[4] Hochella M.F., Mogk D.W., Ranville J., Allen I.C., Luther G.W., Marr L.C., McGrail B.P., Murayama M., Qafoku N.P., Rosso K.M. (2019). Natural, incidental, and engineered nanomaterials and their impacts on the Earth system. Science.

[5] Grandviewresearch.com. Nanomaterials Market Size, Share & Trends Analysis Report By Material (Gold, Silver, Iron, Copper), By Application (Aerospace, Automotive, Medical), By Region, And Segment Forecasts, 2023–2030. https://www.grandviewresearch.com/industry-analysis/nanotechnology-and-nanomaterials-market.

[6] Genchi G., Carocci A., Lauria G., Sinicropi M.S., Catalano A. (2020). Nickel: Human Health and Environmental Toxicology. Int. J. Environ. Res. Public Health.

[7] Guo H., Liu H., Wu H., Cui H., Fang J., Zuo Z., Deng J., Li Y., Wang X., Zhao L. (2019). Nickel Carcinogenesis Mechanism: DNA Damage. Int. J. Mol. Sci..

[8] Zhao Y., Topping T., Bingert J.F., Thornton J.J., Dangelewicz A.M., Li Y., Liu W., Zhu Y., Zhou Y., Lavernia E.J. (2008). High Tensile Ductility and Strength in Bulk Nanostructured Nickel. Adv. Mater..

[9] He X., Zhong W., Au C.T., Du Y. (2013). Size dependence of the magnetic properties of Ni nanoparticles prepared by thermal decomposition method. Nanoscale Res. Lett..

[10] Ishizaki T., Yatsugi K., Akedo K. (2016). Effect of Particle Size on the Magnetic Properties of Ni Nanoparticles Synthesized with Trioctylphosphine as the Capping Agent. Nanomaterials.

[11] Sharma A., Hickman J., Gazit N., Rabkin E., Mishin Y. (2018). Nickel nanoparticles set a new record of strength. Nat. Commun..

[12] Su F., Qiu X., Liang F., Tanaka M., Qu T., Yao Y., Ma W., Yang B., Dai Y., Hayashi K. (2018). Preparation of Nickel Nanoparticles by Direct Current Arc Discharge Method and Their Catalytic Application in Hybrid Na-Air Battery. Nanomaterials.

[13] Phillips J.I., Green F.Y., Davies J.C., Murray J. (2010). Pulmonary and systemic toxicity following exposure to nickel nanoparticles. Am. J. Ind. Med..

[14] Journeay W.S., Goldman R.H. (2014). Occupational Handling of Nickel Nanoparticles: A Case Report. Am. J. Ind. Med..

[15] Kong L., Dong J., Lu W., Wu Y., Liu L., Tang M. (2021). Exposure effects of inhaled nickel nanoparticles on the male reproductive system via mitochondria damage. NanoImpact.

[16] Kong L., Wu Y., Hu W., Liu L., Xue Y., Liang G. (2021). Mechanisms underlying reproductive toxicity induced by nickel nanoparticles identified by comprehensive gene expression analysis in GC-1 spg cells. Environ. Pollut..

[17] Kang G.S., Gillespie P.A., Gunnison A., Moreira A.L., Tchou-Wong K.M., Chen L.C. (2011). Long-term inhalation exposure to nickel nanoparticles exacerbated atherosclerosis in a susceptible mouse model. Environ. Health Perspect..

[18] You D.J., Lee H.Y., Taylor-Just A.J., Linder K.E., Bonner J.C. (2020). Sex differences in the acute and subchronic lung inflammatory responses of mice to nickel nanoparticles. Nanotoxicology.

[19] Almazroo O., Miah M.K., Venkataramanan R. (2017). Drug Metabolism in the Liver. Clin. Liver Dis..

[20] Øie C.I., Mönkemöller V., Hübner W., Schüttpelz M., Mao H., Ahluwalia B.S., Huser T.R., McCourt P. (2018). New ways of looking at very small holes—Using optical nanoscopy to visualize liver sinusoidal endothelial cell fenestrations. Nanophotonics.

[21] Zhang Y.N., Poon W., Tavares A.J., McGilvray I.D., Chan W.C.W. (2016). Nanoparticle-liver interactions: Cellular uptake and hepatobiliary elimination. J. Control Release.

[22] Li X., Wang B., Zhou S., Chen W., Chen H., Liang S., Zheng L., Yu H., Chu R., Wang M. (2020). Surface chemistry governs the sub-organ transfer, clearance and toxicity of functional gold nanoparticles in the liver and kidney. J. Nanobiotechnol..

[23] Macparland S., Tsoi K.M., Ouyang B., Ma X., Manuel J., Fawaz A., Ostrowski M., Alman B., Zilman A., Chan W. (2017). Phenotype Determines Nanoparticle Uptake by Human Macrophages from Liver and Blood. ACS Nano.

[24] Tsoi K.M., MacParland S.A., Ma X.-Z., Spetzler V.N., Echeverri J., Ouyang B., Fadel S.M., Sykes E.A., Goldaracena N., Kaths J.M. (2016). Mechanism of hard-nanomaterial clearance by the liver. Nat. Mater..

[25] Zhou S., Li X., Zhu M., Yu H., Chu R., Chen W., Wang B., Wang M., Zheng L., Chai Z. (2020). Hepatic impacts of gold nanoparticles with different surface coatings as revealed by assessing the hepatic drug-metabolizing enzyme and lipid homeostasis in mice. NanoImpact.

[26] Li J., Chen C., Xia T. (2022). Understanding Nanomaterial-Liver Interactions to Facilitate the Development of Safer Nanoapplications. Adv. Mater..

[27] Magaye R.R., Yue X., Zou B., Shi H., Yu H., Liu K., Lin X., Xu J., Yang C., Wu A. (2014). Acute toxicity of nickel nanoparticles in rats after intravenous injection. Int. J. Nanomed..

[28] Jayaseelan C., Abdul Rahuman A., Ramkumar R., Perumal P., Rajakumar G., Vishnu Kirthi A., Santhoshkumar T., Marimuthu S. (2014). Effect of sub-acute exposure to nickel nanoparticles on oxidative stress and histopathological changes in Mozambique tilapia, Oreochromis mossambicus. Ecotoxicol. Environ. Saf..

[29] Tsuchida D., Matsuki Y., Tsuchida J., Iijima M., Tanaka M. (2023). Allergenicity and Bioavailability of Nickel Nanoparticles Compared to Nickel Microparticles in Mice. Materials.

[30] Ahmad J., Alhadlaq H.A., Siddiqui M.A., Saquib Q., Al-Khedhairy A.A., Musarrat J., Ahamed M. (2015). Concentration-dependent induction of reactive oxygen species, cell cycle arrest and apoptosis in human liver cells after nickel nanoparticles exposure. Environ. Toxicol..

[31] Ajdari M., Ziaee Ghahnavieh M. (2014). Histopathology effects of nickel nanoparticles on lungs, liver, and spleen tissues in male mice. Int. Nano Lett..

[32] Liu F., Chang X., Tian M., Zhu A., Zou L., Han A., Su L., Li S., Sun Y. (2017). Nano NiO induced liver toxicity via activating the NF-kappaB signaling pathway in rats. Toxicol. Res..

[33] Ahamed M., Akhtar M.J., Alhadlaq H.A., Khan M.A., Alrokayan S.A. (2015). Comparative cytotoxic response of nickel ferrite nanoparticles in human liver HepG2 and breast MFC-7 cancer cells. Chemosphere.

[34] Zhu M., Chen H., Zhou S., Zheng L., Li X., Chu R., Chen W., Wang B., Wang M., Chai Z. (2021). Iron oxide nanoparticles aggravate hepatic steatosis and liver injury in nonalcoholic fatty liver disease through BMP-SMAD-mediated hepatic iron overload. Nanotoxicology.

[35] Belew G.D., Jones J.G. (2022). De novo lipogenesis in non-alcoholic fatty liver disease: Quantification with stable isotope tracers. Eur. J. Clin. Investig..

[36] Francque S., Szabo G., Abdelmalek M.F., Byrne C.D., Cusi K., Dufour J.F., Roden M., Sacks F., Tacke F. (2021). Nonalcoholic steatohepatitis: The role of peroxisome proliferator-activated receptors. Nat. Rev. Gastroenterol. Hepatol..

[37] Chen H., Zhou S., Chen W., Zhu M., Yu H., Zheng L., Wang B., Wang M., Feng W. (2023). PEG-GNPs aggravate MCD-induced steatohepatitic injury and liver fibrosis in mice through excessive lipid accumulation-mediated hepatic inflammatory damage. NanoImpact.

[38] Liu H., Lai W., Liu X., Yang H., Fang Y., Tian L., Li K., Nie H., Zhang W., Shi Y. (2020). Exposure to copper oxide nanoparticles triggers oxidative stress and endoplasmic reticulum (ER)-stress induced toxicology and apoptosis in male rat liver and BRL-3A cell. J. Hazard. Mater..

[39] Zambelli B., Ciurli S. (2013). Nickel and human health. Met. Ions Life Sci..

[40] Das K.K., Reddy R.C., Bagoji I.B., Das S., Bagali S., Mullur L., Khodnapur J.P., Biradar M.S. (2018). Primary concept of nickel toxicity—An overview. J. Basic. Clin. Physiol. Pharmacol..

[41] Munoz A., Costa M. (2012). Elucidating the mechanisms of nickel compound uptake: A review of particulate and nano-nickel endocytosis and toxicity. Toxicol. Appl. Pharmacol..

[42] Boey A., Ho H.K. (2020). All Roads Lead to the Liver: Metal Nanoparticles and Their Implications for Liver Health. Small.

[43] Liu L., Lu W., Dong J., Wu Y., Tang M., Liang G., Kong L. (2022). Study of the mechanism of mitochondrial division and mitochondrial autophagy in the male reproductive toxicity induced by nickel nanoparticles. Nanoscale.

[44] Chen H. (2020). Nutrient mTORC1 signaling contributes to hepatic lipid metabolism in the pathogenesis of non-alcoholic fatty liver disease. Liver Res..

[45] Ferré P., Foufelle F. (2010). Hepatic steatosis: A role for de novo lipogenesis and the transcription factor SREBP-1c. Diabetes Obes. Metab..

[46] Chakravarthy M.V., Pan Z., Zhu Y., Tordjman K., Schneider J.G., Coleman T., Turk J., Semenkovich C.F. (2005). “New” hepatic fat activates PPARalpha to maintain glucose, lipid, and cholesterol homeostasis. Cell Metab..

[47] Gong J., Tu W., Liu J., Tian D. (2022). Hepatocytes: A key role in liver inflammation. Front. Immunol..

[48] Ahmed O., Robinson M.W., O’Farrelly C. (2021). Inflammatory processes in the liver: Divergent roles in homeostasis and pathology. Cell Mol. Immunol..

[49] Li J., Sun R., Xu H., Wang G. (2022). Integrative Metabolomics, Proteomics and Transcriptomics Analysis Reveals Liver Toxicity of Mesoporous Silica Nanoparticles. Front. Pharmacol..

[50] Seki E., Schwabe R.F. (2015). Hepatic inflammation and fibrosis: Functional links and key pathways. Hepatology.

[51] Giraldez M.D., Carneros D., Garbers C., Rose-John S., Bustos M. (2021). New insights into IL-6 family cytokines in metabolism, hepatology and gastroenterology. Nat. Rev. Gastroenterol. Hepatol..

[52] Dara L., Ji C., Kaplowitz N. (2011). The contribution of endoplasmic reticulum stress to liver diseases. Hepatology.

[53] Malhi H., Kaufman R.J. (2011). Endoplasmic reticulum stress in liver disease. J. Hepatol..

[54] Ajoolabady A., Kaplowitz N., Lebeaupin C., Kroemer G., Kaufman R.J., Malhi H., Ren J. (2023). Endoplasmic reticulum stress in liver diseases. Hepatology.

[55] Zhang J., Guo J., Yang N., Huang Y., Hu T., Rao C. (2022). Endoplasmic reticulum stress-mediated cell death in liver injury. Cell Death Dis..

[56] Glista-Baker E.E., Taylor A.J., Sayers B.C., Thompson E.A., Bonner J.C. (2012). Nickel nanoparticles enhance platelet-derived growth factor-induced chemokine expression by mesothelial cells via prolonged mitogen-activated protein kinase activation. Am. J. Respir. Cell Mol. Biol..

