# Supplementary material for: Nickel Nanoparticles Induced Hepatotoxicity in Mice via Lipid-Metabolism-Dysfunction-Regulated Inflammatory Injury
Source: Molecules. 2023 Jul 30;28(15):5757. doi: 10.3390/molecules28155757 (PMC10421287; doi:10.3390/molecules28155757)
Supplement: Supplementary file 1 [file molecules-28-05757-s001.zip › molecules-2522375-supplementary.pdf]

Supporting Materials

Nickel Nanoparticles Induced Hepatotoxicity in Mice via Lipid Metabolism Dysfunction Regulated Inflammatory Injury

Shuang Zhou [1,2], Hua Li [1], Hui Wang [1], Rui Wang [1], Wei Song [1], Da Li [1], Changlei Wei [1], Yu Guo [1], Xueying He [1,*] and Yulin Deng [2,*]

1 Beijing Institute of Medical Device Testing, Beijing Center for Testing and Research of Medical Biological Protective Equipment, Beijing 101111, China
2 Beijing Institute of Technology, School of Life Science, Beijing 100081, China
* Correspondence: hexueying@bimt.org.cn (X.H.); deng@bit.edu.cn (Y.D.)

Table S1. The primer sequences used in this work.

Genes	Forward primer sequences (5'-3')	Reverse primer sequences (5'-3')
Tnf-α	ACCCTCACACTCAGATCATC	GAGTAGACAAGGTACAACCC
Il-1β	CTTTGAAGTTGACGGACCC	TGAGTGATACTGCCTGCCTG
Il-6	ACAAGTCGGAGGCTTAATTACACAT	TTGCCATTGCACAACTCTTTT
Ccl-2	CAGGTCCCTGTCATGCTTCT	TCTGGACCCATTCCTTCTTG
Ccl-3	TGTACCATGACACTCTGCAAC	CAACGATGAATTGGCGTGGAA
Ccl-5	GCTGCTTTGCCTACCTCTCC	TCGAGTGACAAACACGACTGC
Ire1α	GAGCAAGCTAACGCCTACTCTGT	CACCATTGAGGGAGAGGCATA
Perk	GGTATTTCAACGCCTGGCTG	GGCCAGTCTGTGCTTTCGTC
Atf6	GTTACTCACCCATCCGAGTTGT	CAACGTCGACTCCCAGTCTTC
Bip	TGCGGCCAAGAACCAACTC	AATGTCTTGGTTTGCCCACCTC
Xbp1s	CAGCAAGTGGTGGATTTGGAAG	TCTTAACTCCTGGTTCTCAACCACA
Atf4	CTGGCCGAGGCTATAAAGGG	TGAAGAGCGCCATGGCTTAG
Chop	AATAACAGCCGGAACCTGAGGA	ACTCAGCTGCCATGACTGCAC
Bax	CCCGAGAGGTCTTTTTCCGAG	CCAGCCCATGATGGTTCTGAT
Bcl-2	CTGGCATCTTCTCCTTCCAG	GACGGTAGCGACGAGAGAAG
Srebp1c	GGAGCCATGGATTGCACATT	GGCCCGGGAAGTCACTGT

Fasn	AGGTGGTGATAGCCGGTATGT	TGGGTAATCCATAGAGCCCAG
Scd1	GCGATACACTCTGGTGCTCA	CCCAGGGAAACCAGGATATT
Pparα	AGAGCCCCATCTGTCCTCTC	ACTGGTAGTCTGCAAAACCAAA
Gadph	AGGTCGGTGTGAACGGATTTG	TGTAGACCATGTAGTTGAGGTCA
